# Carnation Italian Ringspot Virus p36 Expression Induces Mitochondrial Fission and Respiratory Chain Complex Impairment in Yeast

**DOI:** 10.3390/ijms242216166

**Published:** 2023-11-10

**Authors:** Giuseppe Petrosillo, Angelo De Stradis, Domenico Marzulli, Luisa Rubino, Sergio Giannattasio

**Affiliations:** 1 Institute of Biomembranes, Bioenergetics and Molecular Biotechnologies, CNR, Via Amendola 122/O, 70126 Bari, Italy; g.petrosillo@ibiom.cnr.it (G.P.); d.marzulli@ibiom.cnr.it (D.M.); 2 Institute for Sustainable Plant Protection, CNR, UOS Bari, Via Amendola 165/A, 70126 Bari, Italy; angelo.destradis@ipsp.cnr.it

**Keywords:** (+)RNA virus, CIRV, p36, mitochondria, respiratory complex, yeast

## Abstract

Positive-strand RNA virus replication invariably occurs in association with host cell membranes, which are induced to proliferate and rearrange to form vesicular structures where the virus replication complex is assembled. In particular, carnation Italian ringspot virus (CIRV) replication takes place on the mitochondrial outer membrane in plant and yeast cells. In this work, the model host *Saccharomyces cerevisiae* was used to investigate the effects of CIRV p36 expression on the mitochondrial structure and function through the determination of mitochondrial morphology, mitochondrial respiratory parameters, and respiratory chain complex activities in p36-expressing cells. CIRV p36 ectopic expression was shown to induce alterations in the mitochondrial network associated with a decrease in mitochondrial respiration and the activities of NADH–cyt c, succinate–cyt c (C II-III), and cytochrome c oxidase (C IV) complexes. Our results suggest that the decrease in respiratory complex activity could be due, at least in part, to alterations in mitochondrial dynamics. This yeast-based model will be a valuable tool for identifying molecular targets to develop new anti-viral strategies.

## 1. Introduction

Viruses with positive-strand RNA ((+)RNA) genomes represent the vast majority of all known viruses and include the agents of many important human diseases, for example, Chikungunya, Zika, MERS, SARS, and the most recent, COVID-19. A unifying feature of the (+)RNA viruses is the close association of virus replication with intracellular membranes. Independent of the host, whether humans, animals, or plants, all (+)RNA viruses induce specific cell membrane proliferation and rearrangements to form partially closed vesicular invaginations where the virus replication complex is assembled and viral replication takes place. The cell membranes involved in (+)RNA virus infections are of different origins, depending specifically on the virus, and may be derived from the endoplasmic reticulum or the plasma membrane, or from organelles like mitochondria, peroxisomes, vacuoles, chloroplasts, or lysosomes. The common replication mechanisms shared by (+)RNA viruses foster the use of simple plant (+)RNA viruses as models to decipher virus–host cell membrane interactions, which may lead to the identification of host factors co-opted for viral replication and will ultimately facilitate the development of new antiviral strategies.

Members of the plant (+)RNA virus genus *Tombusvirus* (family *Tombusviridae*) normally replicate in association with membranous cytoplasmic structures denoted “multivesicular bodies” (MVBs) regardless of the virus or the host [1,2]. MVBs consist of a main body surrounded by many 80–150 nm globose-to-ovoid vesicles containing double-stranded RNA (dsRNA, [3,4,5]), which are thought to be the sites where virus replication occurs. Typically, MVBs are derived from the progressive invagination of the limiting membrane of peroxisomes in the case of Cymbidium ringspot virus (CymRSV) [1,2] and most tombusviruses [5,6,7]. In contrast, carnation Italian ringspot virus (CIRV) is the only member of the genus *Tombusvirus* known so far to induce the formation of MVBs from deranged mitochondria [2]. 

**Figure 1:**
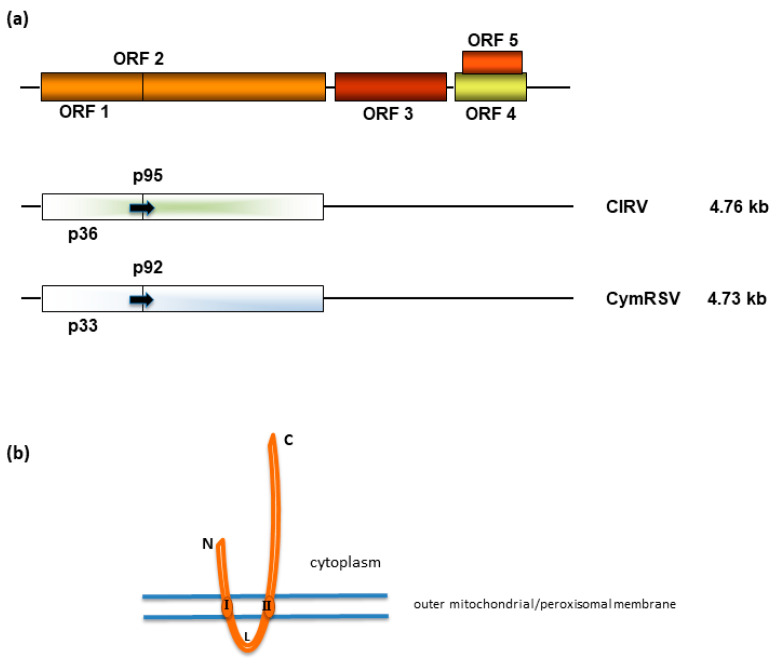
(**a**) Schematic representation of the genome organization (top) and expression of carnation Italian ringspot virus (CIRV) and Cymbidium ringspot virus (CymRSV) genomes (below). Arrows indicate the readthrough of the amber termination codon of CIRV p36 and CymRSV p33, allowing for the synthesis of CIRV p95 and CymRSV p92 replicase proteins, respectively. (**b**) A model of the topology of the insertion of CIRV p36 and CymRSV p33 into the outer mitochondrial or peroxisomal membranes, respectively (drawing not to scale) (see also [15]). N, N-terminus; C, C-terminus; I and II, transmembrane I and transmembrane II domains, respectively; L, loop.

Carnation Italian ringspot virus belongs to the species *Tombusvirus dianthi* in the genus *Tombusvirus* [8]. CIRV isodiametric particles each contain one copy of a single-stranded, (+)RNA genome that is 4.76 kb in size, lacks both a 5′-cap and a 3′-poly(A) tail, and harbors five functional open reading frames (ORFs) [9]. The products of the two 5′-proximal ORFs are synthesized at the early stage of the infection and are both essential for virus replication [10,11,12]. ORF1 codes for the 36 kDa protein (p36), whereas ORF2 is translated after a readthrough of the ORF1 amber stop codon to give a 95 kDa protein (p95) that contains eight conserved motifs (PI–PVIII) of the RNA-dependent RNA polymerase (RdRP) of supergroup II of the (+)RNA viruses [13]. ORF3 codes for the capsid protein (p41), ORF4 codes for the cell-to-cell movement protein (p22), and the nested ORF5 codes for the posttranscriptional gene silencing suppressor (p19) [9] (Figure 1a). It has been shown that the signals responsible for targeting and anchoring the virus replicase to the peroxisomal or the mitochondrial outer membrane are contained in a stretch of *ca*. 200 amino acid in the N-terminal region of the replication-associated CymRSV p33 or CIRV p36 ORF1 products, respectively [14,15]. The signals mainly consist of two hydrophobic transmembrane domains spanning the peroxisomal membrane or the mitochondrial outer membrane, respectively, and in the hydrophilic loop connecting them (Figure 1b) [14,15,16].

Thin-sectioned CIRV-infected cells typically show the presence of cytopathologic structures derived from the progressive invagination of the mitochondrial outer membrane, which leads to the formation of a huge number of vesicles containing fibrillar material consisting of dsRNA, with a short neck connecting the interior of the vesicles to the cytoplasm [4]. The presence of dsRNA fibrils and the association of virus replicase with a mitochondria-enriched cell fraction support the hypothesis that vesicles are the intracellular site of virus replication. In fact, it has been reported that p36 localizes and is stably associated with the outer mitochondrial membrane in plant cells [15,16,17]. 

*Saccharomyces cerevisiae* has been successfully used as an alternative unicellular eukaryotic host to study (+)RNA virus replication and co-opted host factors [18,19,20,21,22]. The replication of both CIRV and CymRSV has been performed in yeast [23,24,25,26] and the membrane-targeting signals present in ORF1 have also been shown to be functional in *S. cerevisiae* cells [16,27,28]. In this host, CIRV p36 localizes to mitochondria in the absence of any other virus product, inducing membrane proliferation and mitochondria alterations in shape [27] and causing a decrease in yeast cell growth rate without promoting cell death [29]. In addition, p36 changed the nature of acetic acid (AA)-induced cell death in yeast by increasing the number of cells dying by necrosis with a concomitant decrease in the number of cells dying by regulated cell death (RCD) in *S. cerevisiae* [29]. 

In view of the key role of p36 in hijacking mitochondrial membranes *en route* to CIRV infection, here we aimed at the functional analysis of the mitochondrial compartment upon p36 expression as well as the identification of possible mitochondrial components/molecules interacting with p36. Thus, given the interaction of p36 with mitochondria in plant and yeast cells, we used the model host *S. cerevisiae* to investigate the possible effects of p36 on mitochondrial structure and function through the determination of mitochondrial networks, mitochondrial respiratory parameters, and respiratory chain complex activities in p36-expressing cells. We show that CIRV p36 ectopic expression induces alterations in the mitochondrial network associated with a decrease in mitochondrial respiration and electron transport chain activity. The yeast model developed and the results obtained will be useful in elucidating the molecular mechanism underlying cellular alterations associated with (+) RNA virus infection.

## 2. Results

### 2.1. CIRV p36 Ectopic Expression Decreased Respiratory Yeast Cell Growth

*S. cerevisiae* cells containing the p36 coding sequence (y-p36) or empty plasmid pYES (y-pYES) were grown for 24 h in the presence of glycerol (as a nonfermentable carbon source) and galactose to induce the expression of p36. Under these growth conditions, the energy demands of the cells are met mainly by mitochondrial oxidative phosphorylation. Cells harboring the p33 coding sequence (y-p33) were also grown under the same conditions as the control. Indeed, when expressed alone in yeast cells, the replication-associated CymRSV p33 specifically localizes on the peroxisomal membrane in the yeast strain UTL-7A in which peroxisome biogenesis can be favored under fixed growth conditions [25,28], and on the endoplasmic reticulum when expressed in yeast strain YHP499, which lacks mature peroxisomes [26].

**Figure 2:**
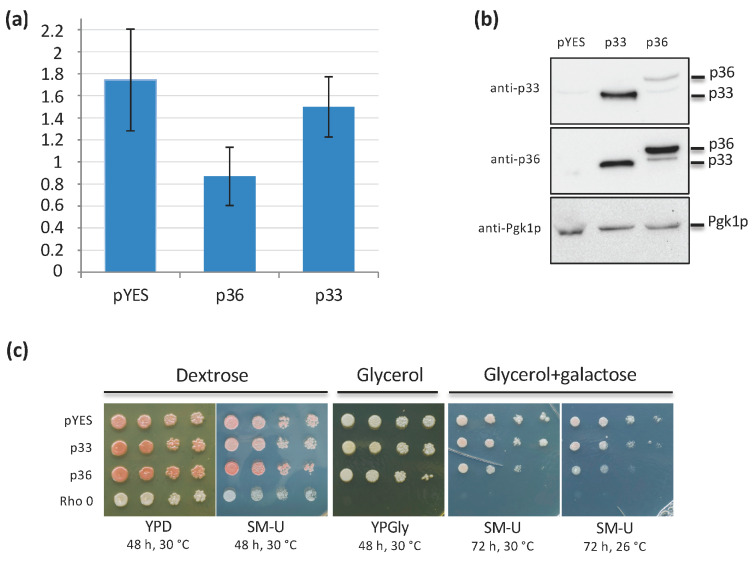
(**a**) CIRV p36 heterologous expression affects respiratory yeast cell growth. y-pYES, y-p36, and y-p33 were analyzed after 24 h growth in a selective minimal medium containing 2% galactose to induce p36 or p33 expression. Cell growth was determined by measuring absorbance at 600 nm. The means of five independent experiments are reported. (**b**) Total protein extraction was performed after 24 h growth in a selective minimal medium containing 2% galactose to induce protein expression. YPH499 yeast cells transformed with empty plasmid pYES and grown under the same conditions served as controls. The electrophoretic mobility of p33, p36, and Pgk1p are indicated on the right. Whole protein extracts were analyzed by immunoblotting and probed with anti-p33 and anti-p36 polyclonal antisera, and with anti-Pgk1p monoclonal antibodies. (**c**) y-pYES, y-p36, or y-p33 grown for 24 h in a selective minimal medium containing 2% galactose to induce p36 or p33 expression were serially diluted and dropped on dextrose-, glycerol-, and glycerol + galactose-containing media. Incubation was at 30 or 26 °C. Rho 0 cells lacking mitochondrial DNA and transformed with plasmid pYES served as controls.

The growth of y-p36 cells was significantly reduced compared with that of y-pYES cells, as previously shown [29], whereas the growth of y-p33 cells was the same as that of y-pYES cells, as determined spectrophotometrically (Figure 2a). To verify p33 and p36 expression, whole cell lysates were prepared from y-pYES, y-p36, and y-p33 cells and subjected to Western blot analysis using polyclonal antisera raised against p36 [16] or p33 [30]. Immunoblotting analysis with anti-p33 antibody allowed the detection of an immunoreactive band corresponding to p33 molecular weight only in y-p33, a faint band corresponding to p36 molecular weight in y-p36 cells, and no immunoreactive bands in y-pYES cells (Figure 2b). On the other hand, anti-p36 antibodies allowed the detection of either p33 or p36 immunoreactive bands in y-p33 and y-p36 cells, respectively. Both p36 and p33 proteins were expressed at the same level, as compared with the yeast cytosolic protein Pgk1p (Figure 2b). Further analyses were carried out using only anti-p36 antibodies.

Altogether, these results are in agreement with our previous observation [29] and confirm the specific effect of p36 expression on cell growth. Indeed, the expression of another membrane-interacting protein, such as p33, was ineffective even though its role parallels p36 function in viral replication.

To investigate whether mitochondrial metabolism has a role in cell growth inhibition, the same cultures were serially diluted and plated on YP media containing dextrose (YPD) or glycerol (YPGly) as carbon sources to check for cell respiratory competency. Whereas yeast cells growing on YPD had similar growth, y-p36 cells showed a growth decrease on YPGly compared with y-pYES or y-p33 (Figure 2c). Similarly, all cells placed on a synthetic selective medium (SM-U) containing dextrose showed no difference in growth. A striking decrease was observed in the growth of p36-expressing cells cultivated on selective media containing glycerol and galactose (to maintain viral protein expression) compared with pYES and p33-expressing control cells (Figure 2c). The difference was even greater when plates were incubated at 26 °C (Figure 2c), since at this temperature viral replicase protein expression is favored [27]. Thus, p36 expression negatively affects yeast cell growth on a non-fermentable carbon source, suggesting the involvement of mitochondria in this effect.

### 2.2. The Mitochondrial Network Is Affected by p36 Expression in Yeast Cells

**Figure 3:**
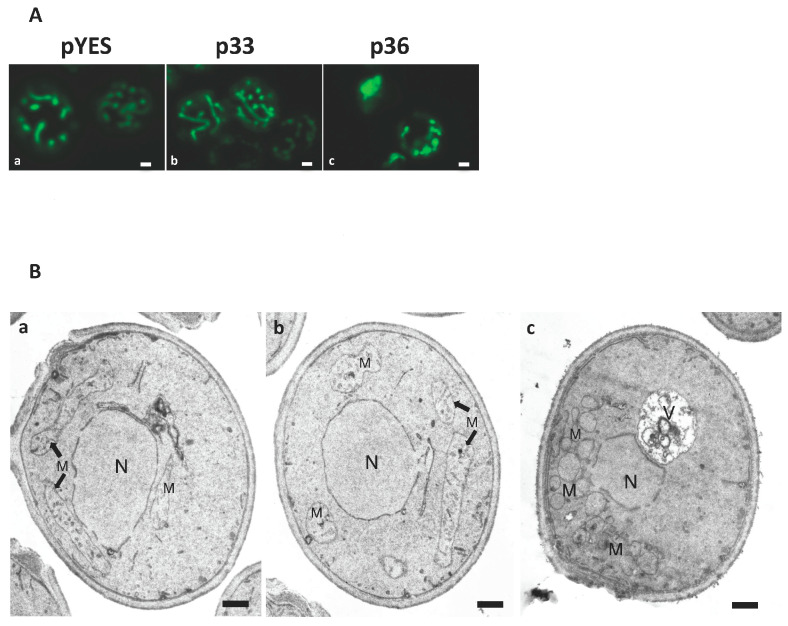
Mitochondrial fission occurs in p36-expressing yeast cells. (**A**) YPH499 cells co-transformed with pYX232-mtGFP and plasmid pYES, empty or containing CIRV p36 or CymRSV p33 sequences, respectively, were grown for 24 h in a selective minimal medium containing 2% galactose to induce p36 or p33 expression and analyzed using confocal microscopy. Single optical sections showed the distribution of the mitochondrial network in control cells transformed with pYES (a) or in cells expressing p33 (b). When CIRV p36 was expressed, mtGFP fluorescence was restricted to defined regions in the cytoplasm (c). Bar: 1 μM. (**B**) Electron micrographs of *Saccharomyces cerevisiae* YPH499 cells transformed with empty plasmid pYES (a) or expressing CymRSV p33 (b) or CIRV p36 (c) replicase proteins. Yeast cells were fixed with aldehydes, post-fixed with sodium permanganate, and embedded in Spurr resin. pYES-transformed (a) and p33-expressing (b) control cells showed long, branched mitochondria with visible cristae, whereas p36 expression (c) increased the number of small, misshaped mitochondria that were clustered and had no visible cristae. M, mitochondria; N, nucleus; V, vacuole. Bar: 100 nm.

In order to investigate mitochondrial function upon p36 expression, we first analyzed the effect of CIRV p36 expression on the mitochondrial morphology. To this aim, y-p36, y-p33, and y-pYES cells were co-transformed with plasmid pYX232-mtGFP containing a DNA sequence coding for a mitochondrial pre-sequence fused to the GFP coding region (mtGFP) under the control of the constitutive TPI promoter [31]. Transformed cells were grown for 24 h at 26 °C in the appropriate selective synthetic medium containing galactose to induce the expression of p36 or p33, then observed under a confocal microscope. The characteristic branched tubular mitochondrial network was observed in both y-pYES and y-p33 cells, whereas y-p36 cells showed a punctuated mitochondrial morphology indicative of mitochondrial network fission (Figure 3A).

To gain a deeper insight into the nature of the fluorescent aggregates, electron microscope analysis was carried out on yeast cells expressing mtGFP alone or together with p33 or p36. y-pYES and y-p33 control cells expressing mtGFP showed the presence of few long, tubular mitochondria with well-developed cristae, arranged all around the cell (Figure 3B, panels a and b), corresponding to the fluorescent tubular network (Figure 3A). y-p36 cells co-expressing mtGFP contained a huge number of small, misshapen mitochondria with no cristae, mostly concentrated in regions of the cells and forming aggregates (Figure 3B, panel c), which likely correspond to the fluorescent structures observed under the confocal microscope (Figure 3A). It can be concluded that p36 expression causes mitochondrial remodeling involving organelle division [32].

### 2.3. Mitochondrial Proteins Are Not Affected by p36 Expression

**Figure 4:**
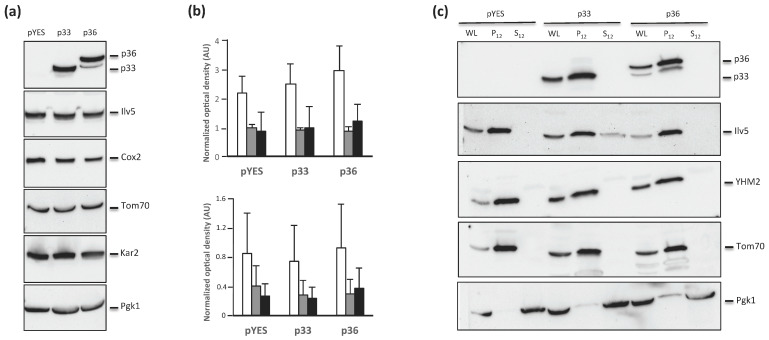
(**a**) Western blot analysis of whole cell lysates showing p33 or p36 expression. Total protein extraction was performed after 24 h of growth in a selective minimal medium containing 2% galactose to induce protein expression. YPH499 yeast cells transformed with empty plasmid pYES and grown under the same conditions served as controls. The positions of p33, p36, Ilv5, Cox2, Tom70, Kar2, and Pgk1 are indicated on the right. Whole protein extracts were analyzed by immunoblotting and probed with anti-p36, anti-Ilv5, anti-Tom70, and anti-Kar2 polyclonal antisera, and with anti-Cox2 and anti-Pgk1 monoclonal antibodies. (**b**) The histograms report the results of the densitometric analysis carried out using Chemidoc (BioRad) of immunoreactive bands of Ilv5, Cox2, and Tom70 (white, gray, and black bars, respectively) normalized with antibodies against Pgk1 (upper plot) or Kar2 (lower plot). Reported values are the means of three different experiments with standard deviations. (**c**) Western blot analysis of whole cell lysates (WL), membrane enriched (P12), and soluble (S12) fractions obtained from yeast cells transformed with the empty pYES plasmid or expressing p33 or p36. Protein extractions were performed after 24 h of growth in a selective minimal medium containing 2% galactose to induce protein expression. The positions of p33, p36, Ilv5, YHM2, Tom70, and Pgk1 are indicated on the right. Whole protein extracts were analyzed by immunoblotting and probed with anti-p36, anti-Ilv5, anti-YHM2, and anti-Tom70 polyclonal antisera, and with anti-Cox2 and anti-Pgk1 monoclonal antibodies.

In view of reshaping the mitochondria upon p36 expression (Figure 3), we wondered whether p36 could also affect the biogenesis of mitochondrial proteins. To this aim, we analyzed by immunoblotting the levels of Ilv5, Cox2, and Tom70 proteins localized in the matrix and the inner and the outer mitochondrial membranes, respectively, in y-p36 whole cell lysate as well as in y-p33 and y-pYES cells as controls. The presence of endoplasmic reticulum chaperon Kar2 and cytosolic Pgk1 was also analyzed using specific antibodies as controls. As expected, two immunoreactive bands corresponding to p36 and p33 were detected with anti-p36 antibodies in y-p36 and y-p33 cell lysates, respectively, with similar intensities compared with Pgk1 (Figure 4a). The levels of Ilv5, Tom70, and Cox2 were not affected by either p36 or p33 expression compared with y-pYES cell lysates (Figure 4a,b). These results show that neither p36 nor p33 expression altered the biogenesis of mitochondrial or endoplasmic reticulum membrane proteins in yeast cells.

We then confirmed that in our experimental setup, both p36 and p33 were mostly localized to the membrane-enriched cell fraction, as previously reported [15,27,29]. To do this, spheroplasts were obtained from y-p36, y-p33, or y-pYES cells to prepare membrane-enriched and soluble fractions, as in [27]. The same antibodies against marker proteins of mitochondrial compartments were used, except that anti-Yhm2 replaced anti-Cox20 as a marker of the inner mitochondrial membrane. Immunoblot analysis of the membrane-enriched fraction showed the presence of p36 and p33 together with the immunoreactive bands of Ilv5, Yhm2, and Tom70, whereas no p36- or p33-immunoreactive bands were observed in the soluble fractions in which only Pgk1 was detected (Figure 4b). Detection of trace amounts of Ilv5 in the soluble fraction of y-p33 cells is indicative of the partial breakage of mitochondria during cell fractionation [29].

### 2.4. Heterologous Expression of p36 Decreases Mitochondrial Oxygen Consumption by Inhibiting Respiratory Chain Complex Activities in Yeast Cells

We observed that p36 expression affects yeast cell viability on a non-fermentable carbon source and causes mitochondrial remodeling without changes in mitochondrial protein content. These results prompted us to give an insight into the effect of p36 expression on mitochondrial function. To this aim, we measured mitochondrial respiratory parameters in y-p36 and y-p33 cells, and y-pYES cells as controls, after 24 h of growth in a synthetic selective medium containing glycerol and galactose.

**Figure 5:**
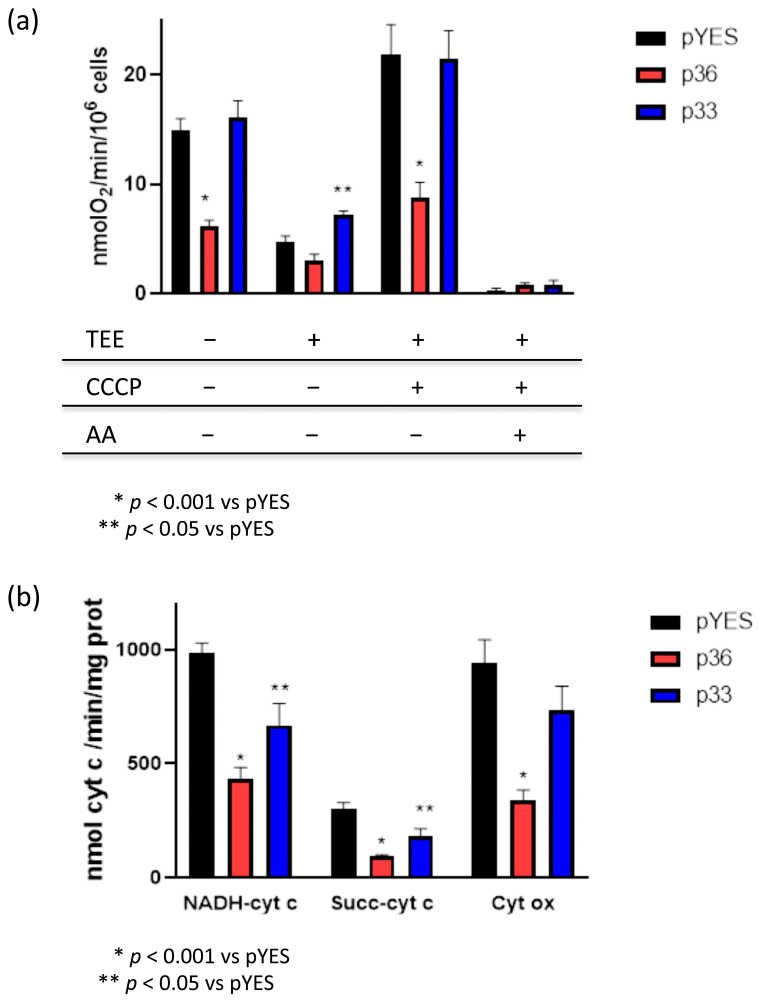
Mitochondrial function in yeast cells and spheroplasts. (**a**) The oxygen consumption rate was measured in intact pYES, p36, and p33 yeast cells. Different stages of the oxidative phosphorylation process were measured in the presence of triethyltin bromide (TEE) to inhibit ATP synthase, carbonyl cyanide 3-chlorophenylhydrazone (CCCP) to increase proton permeability across the inner mitochondrial membrane, and antimycin A (AA) to inhibit mitochondrial respiration at the level of complex III. Each value represents the mean ± SE of six separate experiments. * *p* < 0.001 vs. pYES. (**b**) Respiratory chain complex activities in pYES, p36, and p33 yeast spheroplasts. NADH–cytochrome c oxidoreductase (NADH–cyt c), Succinate–cytochrome c reductase (Succ–cyt c), and Cytochrome c oxidase (Cyt ox) activities were measured as described in the Materials and Methods section. Each value represents the mean ± SE of six separate experiments. * *p* < 0.001 vs. pYES; ** *p* < 0.05 vs. pYES.

To modulate different stages of the oxidative phosphorylation process, the following compounds were added to yeast cells: triethyltin bromide (TEE) to inhibit ATP synthase [33], carbonyl cyanide 3-chlorophenylhydrazone (CCCP) to increase proton permeability across the inner mitochondrial membrane (IMM), and antimycin A (AA) to inhibit mitochondrial respiration at the level of complex III. As shown in Figure 5a, the oxygen consumption rate under basal conditions, which is mainly driven by proton flux through mitochondrial ATP synthase and by the maximal oxygen consumption rate in the presence of the uncoupler CCCP that promotes proton transport across the IMM and drives mitochondria to consume more oxygen to replenish the proton gradient was significantly reduced in y-p36 cells compared with y-pYES control cells. Respiratory rate in the presence of TEE, which reflects oxygen consumption predominantly due to proton leak across the IMM, did not show significant differences between p36-expressing cells and control cells (Figure 5a). Therefore, the expression of p36 in yeast cells affects mitochondrial respiration. Interestingly, expression of ER-targeted p33 did not lead to significant alterations in mitochondrial respiratory parameters compared with y-pYES, with the exception of oxygen consumption in the presence of TEE, showing a p33-induced increase in proton leak (Figure 5a). Oxygen consumption rate in all three types of cells appeared to be exclusively due to mitochondrial respiration since it was almost completely inhibited by the addition of AA.

To investigate how the association of p36 with mitochondria induces alterations in mitochondrial respiration, the activities of IMM respiratory chain complexes were measured. To this aim, we chose to use spheroplasts, which are physiologically normal and comparable to whole cells, and contain functional mitochondria but have been deprived of the cell wall [33]. Spheroplasts were prepared from y-p36 and y-p33 cells, as well as from y-pYES cells, and NADH–cyt c, succinate–cyt c (C II-III), and cytochrome c oxidase (C IV) activities were measured after freeze–thawing. As shown in Figure 5b, the electron transport activities from NADH to cytochrome c and from succinate to cytochrome c were significantly diminished in spheroplasts prepared from p36-expressing cells compared with control spheroplasts. Furthermore, cytochrome c oxidase activity also showed a dramatic decrease in y-p36 with respect to y-pYES. Spheroplasts obtained from p33-expressing cells showed a significant attenuation of NADH—cyt c oxidoreductase and Succinate—cyt c reductase activities compared with y-pYES, but much lower than in the case of p36-expressing cells, while no significant effects were found in the activity of cytochrome c oxidase.

**Figure 6:**
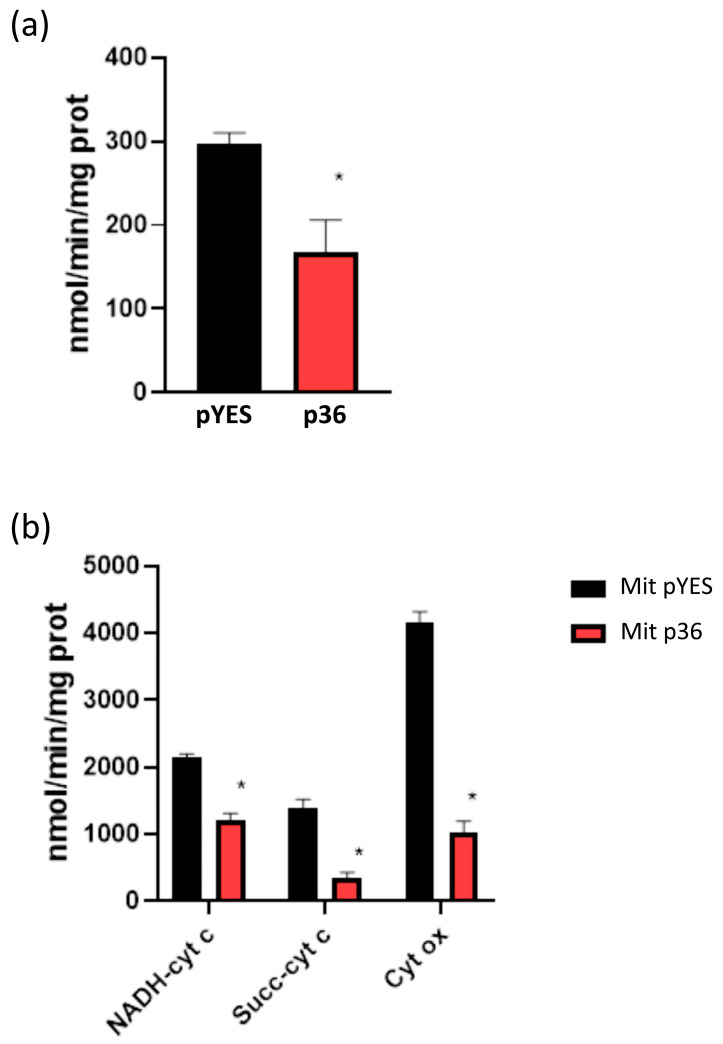
State 3 respiration and respiratory chain complex activities in mitochondria isolated from pYES and p36 yeast cells. (**a**) Respiration rate in freshly isolated mitochondria from pYES and p36 yeast cells. ADP-stimulated mitochondrial respiration was measured using succinate as a substrate, as described in the Materials and Methods section. Each value represents the mean ± SE of three separate experiments. * *p* < 0.05 vs pYES. (**b**) Mitochondrial respiratory chain complex activities in mitochondria isolated from pYES and p36 yeast cells. NADH–cytochrome c oxidoreductase (NADH–cyt c), Succinate–cytochrome c reductase (Succ–cyt c), and Cytochrome c oxidase (Cyt ox) activities were measured as described in the Materials and Methods section. Each value represents the mean ± SE of three separate experiments. * *p* < 0.001.

The observation of decreased mitochondrial respiration and respiratory complex activities might be due to a decrease in the number of functional mitochondria caused by p36/p33 expression even in the presence of similar amounts of mitochondrial proteins. To check this hypothesis, state three respiration and activities of electron transport chain complexes were measured in isolated mitochondria. Since almost all bioenergetic parameters measured did not significantly differ between p33-expressing cells and cells transformed with the empty plasmid, we chose not to include mitochondria isolated from y-p33 cells in the following experiments. Respiratory activity of freshly isolated mitochondria measured in the presence of succinate, which drives the respiratory flux through complex II (succinate dehydrogenase), as a substrate and ADP to stimulate respiration are reported in Figure 6a. A significant decrease in the rate of ADP-stimulated respiration (state three) was observed in mitochondria extracted from p36-expressing cells compared with mitochondria from control cells. 

The activities of the respiratory complexes were also measured in mitochondria obtained from p36-expressing and control cells. NADH–cyt c, Succinate–cyt c (C II-III), and cytochrome c oxidase (C IV) activities were dramatically decreased in mitochondria isolated from y-p36 cells compared with mitochondria isolated from control cells (Figure 6b). These results indicate that the electron transfer activities through yeast NADH dehydrogenases [33] and C III, through C II and C III, and from cytochrome c to oxygen were severely impaired. Thus, p36 expression in yeast cells is associated with alterations in mitochondrial respiratory parameters and respiratory chain complex activities as measured in both spheroplasts and isolated mitochondria.

Overall, our results, in association with the observation that p36 expression did not affect mitochondrial protein content (see Figure 4a), suggest that the altered mitochondrial respiratory activity is not due to a decrease in the number of functional mitochondria per cell but rather to p36-dependent decrease in specific activities of the mitochondrial respiratory chain complexes.

## 3. Discussion

The interaction between CIRV and mitochondria and the ultrastructure of CIRV replication in infected cells have been subjects of intense investigations. Mitochondria represent the CIRV intracellular replication site in plant and yeast cells [4,16]. The viral determinants for mitochondrial targeting of the CIRV replication complex, including p36 motifs and topology, have been characterized in detail [14,15,16]. However, limited data are available on the effects of CIRV p36 on host cell homeostasis [29]. Here, we used the yeast model host to analyze the mitochondrial function in the presence of p36 expression. In agreement with our previous findings [29], we observed significantly reduced growth of p36-expressing yeast cells. The specificity of the p36 effect is clearly demonstrated by the lack of effect on cell growth by CymRSV p33, an accessory replication protein with the same functions as p36 but is targeted to ER membranes in the YPH499 yeast strain. Thus, the negative effect on cell growth is specifically caused by p36 ectopic expression and not by a p36-committed unspecific effect on subcellular organelles. In fact, y-p33 cells did not show any significantly reduced growth compared with control cells, despite p33 expression in yeast cells inducing the proliferation of ER membranes under the same culture conditions [29]. In the present investigation, we demonstrated that several parameters of mitochondrial bioenergetics such as mitochondrial oxygen consumption and respiratory chain complex activities were significantly diminished in p36-expressing yeast cells. We found that basal respiration, as well as maximal respiration (in the presence of uncoupler), were dramatically reduced in p36-expressing cells compared with pYES cells. These alterations were associated with alterations in mitochondrial respiratory chain complex activities in yeast spheroplasts. Several reports have revealed direct connections between membranes of different cellular organelles, particularly between the ER and mitochondria [34]. These connections maintain the normal function of both organelles [35]. Thus, increased proton leak as well as changes in NADH—cyt c and Succinate—cyt c activities in p33-expressing cells could be ascribed, at least in part, to alterations in the molecular tether between these two organelles.

The p36-induced mitochondrial dysfunction could be due to changes in the content of mitochondria per cell. However, no appreciable changes in the masses of certain mitochondrial proteins, measured using immunoblotting analysis, were detected in the yeast preparations. Furthermore, alterations in mitochondrial respiration and mitochondrial electron transport chain activity were found in both yeast cells and mitochondria isolated from p36-expressing cells. These results suggest that the content of mitochondria remains essentially constant, while mitochondrial function gets impaired in p36-expressing yeast cells. It is accepted that the balance between mitochondrial fusion and fission contributes to the maintenance of mitochondrial function [32,36,37]. In this respect, our data clearly show that p36-dependent mitochondrial division and network fission are linked to respiratory complex impairment (Figure 3, Figure 5 and Figure 6), probably due to a change in lipid structure/composition. Indeed, the p36-mediated alterations in the mitochondrial network may be an effect of the well-known capacity of p36 to co-opt lipids to the viral replication sites [38] mediated by host factors like Fis1 and ORP family member proteins, which are needed for the mitochondrial fission/fusion process and to form membrane contact sites, respectively [39,40]. Phospholipids are the main building blocks and play multiple roles in biological membranes. They modulate the functional properties of membrane-associated activities and provide a matrix for the assembly and function of the respiratory chain complexes [41,42].

Evidence from functional and structural studies supports the existence of a supramolecular assembly of the individual components of the mitochondrial electron transport chain in a higher-order structure referred to generically as supercomplex or respirasome. This supramolecular organization seems to provide more efficient electron transfer, preventing excessive ROS generation. Appropriate mitochondrial membrane lipid composition is required for association, stabilization, and functioning of individual complexes and their organization into supercomplexes [43]. Thus, the decrease in respiratory complex activity could be due, at least in part, to membrane lipid reconfiguration.

Our findings identified mitochondrial respiratory complexes as key p36 interactors during viral replication. However, p36 is anchored to the outer mitochondrial membrane through two transmembrane domains (see Figure 1) [15,16,17], suggesting that p36 can affect mitochondrial contact sites, in line with [39].

It has been demonstrated that host glycolytic and fermentation pathway enzymes are recruited to the virus replication sites due to both p36 and p33 activities [44,45]. Moreover, the co-opted glycolytic and fermentative enzymes allow the production of ATP locally, which serves tombusvirus replication [44]. It has been proposed that the co-opted fermentation pathway in the tombusvirus replication compartment corresponds to a metabolic reprogramming like the one induced in cancer cells, i.e., aerobic glycolysis, to provide new metabolic compounds for the proliferation of cellular components. 

This, together with our findings showing that decreased respiratory cell growth in p36-expressing yeast cells is associated with strong inhibition of mitochondrial respiration, suggests a possible metabolic shift towards fermentation. This hints at developing new antiviral strategies to inhibit the molecular process of subversion of the fermentative enzymes.

The modulation of mitochondrial morphology and functions by several viruses, including (+)RNA viruses, has been extensively studied, particularly in the frame of the mechanisms of immune response to viral infections [46]. Different from other (+)RNA virus proteins such as poliovirus 2B and hepatitis virus C NS3/4A, which have a role in cell death induction associated with electron transport chain impairments, CIRV p36 does not cause cell death [29], confirming a mechanism of interaction with mitochondria that can preserve host cell survival for productive viral replication.

## 4. Materials and Methods

### 4.1. Yeast Strain, Plasmids, Transformation, Culture Conditions, and Cell Fractionation

*S. cerevisiae* strain YPH499 (MATa *ura3-52 lys2-801 ade2-101 trp1-*Δ*63 his3*-Δ*200 leu2*-Δ*1*, [47]) was used in all experiments. Yeast cells were transformed using the lithium acetate–polyethylene glycol method [48] with plasmid pYES (Invitrogen, Whaltham, MA, USA), empty or containing the CIRV p36 [27,29] or the CymRSV p33 (Rubino and Russo, unpublished) sequences cloned under the control of the galactose-inducible *GAL1* promoter. Plasmid pYX232-mtGFP, containing a mitochondria-targeted green fluorescent protein (mtGFP) sequence under the control of the constitutive triose phosphate isomerase (*TPI*) promoter [31] was a kind gift from Dr. Ralf Braun, Universität Bayreuth, Bayreuth, Germany. Transformed yeast cells were grown and maintained on synthetic selective medium (SM) lacking uracil (SM-U) or both uracil and tryptophan (SM-UT), depending on the transformant, and containing 2% dextrose. For protein expression, yeast cells were grown in the appropriate liquid synthetic selective medium containing 2% dextrose, then 3% glycerol and 0.1% dextrose at 30 °C for 9 and 16 h, respectively, and finally, at 26 °C for 20–24 h in SM-U or SM-UT containing 3% glycerol and 2% galactose [29].

Yeast cell growth was estimated spectrophotometrically at 600 nm. Serial decimal dilutions of 0.25 OD_600_/mL cultures were placed on rich dextrose or glycerol plates and SM-U plates containing 2% dextrose or 3% glycerol and 2% galactose. Incubation was at 30 or 26 °C for 48 or 72 h. 

Mitochondria were extracted from 250–400 OD_600_ yeast cells grown in galactose-containing induction media for 24 h at 26 °C following the procedure in [49]. 

### 4.2. Protein Extraction and Western Blot Analysis

Yeast cells (2–3 OD_600_) were sedimented at 4000 rpm for 2 min, washed with sterile distilled water, pelleted, and lysed in 0.1 N NaOH at room temperature for five minutes according to [50]. Whole-cell lysates were separated in 12% sodium dodecyl sulfate-polyacrylamide gel electrophoresis (SDS-PAGE) [51], then transferred to polyvinylidene difluoride (PVDF, 0.2 µm) membranes (BioRad, Hercules, CA, USA) using a TransBlot Turbo transfer system (BioRad) following the manufacturer’s instructions. Western blot analysis was carried out to probe the membranes with specific polyclonal antisera raised against p33 [30] or p36 [16]. In addition, a monoclonal anti-phosphoglycerate kinase (anti-Pgk1p, Molecular Probes, Eugene, OR, USA), a polyclonal anti-ER chaperone Kar2 (anti-Kar2p, Santa Cruz Biotechnology, Dallas, TX, USA), a polyclonal anti-acetohydroxyacid reductoisomerase, a monoclonal anti-cytochrome c oxidase subunit II (anti-Ilv5p and anti-Cox2p), and polyclonal antisera raised against Yhm2p and anti-Tom70p were used. Horseradish peroxidase-conjugated anti-rabbit and anti-mouse secondary antibodies (Thermo Scientific, Waltham, MA, USA) were employed for detection with Clarity Western ECL Substrate (BioRad) in a ChemiDoc Imaging System (BioRad).

Prior to respiratory chain complex activity analysis, yeast cells (*ca*. 20 OD_600_) were spheroplasted according to [52]. For protein extraction, aliquots corresponding to 2 OD_600_ cells were sedimented and resuspended in a 50 µL loading buffer [50]. Alternatively, spheroplasts were lysed and proteins were fractionated to obtain a pellet and a supernatant fraction [27,52], denatured in Laemmli loading buffer [32,51], separated using 12% SDS-PAGE, transferred to PVDF membranes, and subjected to Western blot analysis as described above.

### 4.3. Confocal and Electron Microscopy

For confocal microscopy, yeast cells were immobilized on glass slides by mixing one volume (5 µL) of cells with one volume of 2% low melting point agarose in water kept at 45 °C, covered with a cover-slip, and sealed. Samples were observed under a Leica TCS SP5 confocal microscope with excitation at 488 nm and emission at 500–600 nm. Leica Application Suite Advanced Fluorescence (LAS AF) software 2.2.1, and ImageJ software 1.8.0 were utilized for acquisition and image processing.

For electron microscopy, cells (5–10 OD_600_) grown in the appropriate SM medium containing 3% glycerol and 2% galactose were fixed with 2% (*v*/*v*) glutaraldehyde in 0.1 M cacodylate buffer (pH 7.2), then washed with the same buffer, mildly treated with lyticase, post-fixed with 4% potassium permanganate, and bulk stained with uranyl acetate. Then, cells were dehydrated with ethanol and embedded in Spurr’s resin. Ultrathin sections were stained with lead citrate and observed under a Philips Morgagni 282D electron microscope.

### 4.4. Oxygen Consumption

Mitochondrial respiration was analyzed using a Clark-type oxygen electrode (Oxygraph, Hansatech Instruments Ltd., Pentney, UK). Oxygen consumption in yeast cells was measured at 30 °C in 0.5 mL of fresh culture medium using a concentration of 2 × 10^6^ cells/mL. Mitochondrial respiratory parameters were determined in intact cells by measuring the oxygen consumption rate during the sequential addition of the following inhibitors and uncoupler: 200 μM triethyl-tin (TEE) [33,53], 10 μM CCCP, and 8 μM Antimycin A (AA). Respiratory rates are defined as nmol O_2_/min/1 × 10^6^ cells.

Respiratory activity in freshly isolated mitochondria was detected at 28 °C in 0.5 mL of a medium composed of 1 M sorbitol, 0.5 mM EGTA, 2 mM MgCl_2_, 1.7 mM NaCl, 10 mM potassium phosphate, and 0.1% bovine serum albumin, pH 6.8 [33], in the presence of 10 mM succinate as substrate. State 3 respiration was induced by the addition of 200 μM ADP. Respiratory rates are defined in nmol O^2^/min/mg protein.

### 4.5. Respiratory Chain Complex Activities

Respiratory chain complex activities were measured spectrophotometrically at 30 °C in spheroplasts or isolated mitochondria suspended in 20 mM phosphate buffer (pH 7.5) and subjected to 3 cycles of freeze-thaw to disrupt membrane integrity [54].

#### 4.5.1. NADH–Cytochrome c Oxidoreductase

NADH–cytochrome c oxidoreductase (NADH–cyt c) activity was measured by following the increase in absorbance of oxidized cytochrome c at 550 nm in 1 mL of medium consisting of 50 mM phosphate buffer (pH 7.5), 1 mM azide, and 50 μM oxidized cytochrome c in the presence of 50 μg of spheroplast protein or 20 μg of mitochondrial protein [54]. After 2 min of incubation, the reaction was started by the addition of 0.6 mM NADH. The reaction was stopped by the addition of 5 μM Mixothiazol. The activity was calculated using an extinction coefficient of 18.5 mM^−1^ cm^−1^ for reduced cytochrome c. The activity of NADH–cytochrome c oxidoreductase is expressed as nmol of cytochrome c reduced/min/mg protein. The Mixothiazol-insensitive rate of cytochrome c reduction was measured and subtracted.

#### 4.5.2. Succinate–Cytochrome c Reductase (Complex II + III)

Succinate–cytochrome c reductase (Succ—cyt c) activity was measured by following the increase in absorbance of oxidized cytochrome c at 550 nm in 1 mL of medium consisting of 50 mM phosphate buffer (pH 7.5), 1 mM azide, 0.13 mM ATP, and 50 μM oxidized cytochrome c in the presence of 50 μg of spheroplast protein or 20 μg of mitochondrial protein [54]. After 5 min of incubation, the reaction was started by the addition of 8 mM Succinate. The reaction was stopped by the addition of 5 μM Mixothiazol. The activity was calculated using an extinction coefficient of 18.5 mM^−1^cm^−1^ for reduced cytochrome c. The activity of Succinate–cytochrome c reductase is expressed as nmol of cytochrome c reduced/min/mg protein. The Mixothiazol-insensitive rate of cytochrome c reduction was measured and subtracted.

#### 4.5.3. Cytochrome c Oxidase (Complex IV)

Cytochrome c oxidase (Cyt ox) activity was detected by following the decrease in absorbance of reduced cytochrome c at 550 nm in 1 mL of medium consisting of 25 mM phosphate buffer (pH 7), 0.75 mM lauryl maltoside, and 50 μM of reduced cytochrome c. After 2 min of incubation, the reaction was started by the addition of 50 μg of spheroplast protein or 20 μg of mitochondrial protein. The reaction was stopped by the addition of 1 mM azide. The activity was calculated using an extinction coefficient of 18.5 mM^−1^ cm^−1^ for reduced cytochrome c. The specific activity of the enzyme is expressed as nmol of cytochrome c oxidized/min/mg protein. The azide-insensitive rate of cytochrome c oxidation was measured and subtracted.

## Data Availability

The data presented in this study are available on request from the corresponding authors.
